# 4,4′-(1,1,1,3,3,3-Hexafluoro­propane-2,2-diyl)dibenzoic acid

**DOI:** 10.1107/S1600536810017745

**Published:** 2010-05-19

**Authors:** Long Tang, Ya-Pan Wu, Feng Fu, Zhu-Lian Zhang, Dan Zheng

**Affiliations:** a Department of Chemistry and Chemical Engineering, Shaanxi Key Laboratory of Chemical Reaction Engineering, Yan’an University, Shaanxi 716000, People’s Republic of China

## Abstract

In the title compound, C_17_H_10_F_6_O_4_, the two benzene rings are twisted with respect to each other, making a dihedral angle of 67.43 (12)°. In the crystal, adjacent mol­ecules are linked by O—H⋯O and C—H⋯F hydrogen bonding, forming a wave-like layered supra­molecular structure.

## Related literature

For the use of bibenzoic acids as bridging ligands for the synthesis of novel solid-state architectures, see: Zou *et al.* (2007 ▶). For the structures of related dibenzoic acid compounds, see: Potts *et al.* (2007 ▶); Lian *et al.* (2007 ▶).
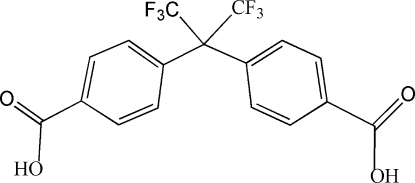


## Experimental

### 

#### Crystal data

C_17_H_10_F_6_O_4_
*M* *_r_* = 392.25
Monoclinic, 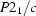

*a* = 7.7523 (16) Å
*b* = 13.381 (3) Å
*c* = 16.134 (3) Å
β = 102.294 (4)°
*V* = 1635.2 (6) Å^3^
*Z* = 4
Mo *K*α radiation
μ = 0.16 mm^−1^
*T* = 293 K
0.35 × 0.20 × 0.18 mm

#### Data collection

Bruker SMART CCD diffractometer
Absorption correction: multi-scan (*SADABS*; Sheldrick, 1996 ▶) *T*_min_ = 0.963, *T*_max_ = 0.972
8113 measured reflections
2904 independent reflections
1339 reflections with *I* > 2σ(*I*)
*R*_int_ = 0.053

#### Refinement

*R*[*F* ^2^ > 2σ(*F* ^2^)] = 0.040
*wR*(*F* ^2^) = 0.071
*S* = 1.00
2904 reflections
246 parameters
H-atom parameters constrained
Δρ_max_ = 0.17 e Å^−3^
Δρ_min_ = −0.19 e Å^−3^

### 

Data collection: *SMART* (Bruker, 1997 ▶); cell refinement: *SAINT* (Bruker, 1997 ▶); data reduction: *SAINT*; program(s) used to solve structure: *SHELXS97* (Sheldrick, 2008 ▶); program(s) used to refine structure: *SHELXL97* (Sheldrick, 2008 ▶); molecular graphics: *ORTEP-3 for Windows* (Farrugia, 1997 ▶); software used to prepare material for publication: *WinGX* (Farrugia, 1999 ▶).

## Supplementary Material

Crystal structure: contains datablocks I, global. DOI: 10.1107/S1600536810017745/xu2737sup1.cif

Structure factors: contains datablocks I. DOI: 10.1107/S1600536810017745/xu2737Isup2.hkl

Additional supplementary materials:  crystallographic information; 3D view; checkCIF report

## Figures and Tables

**Table 1:** Hydrogen-bond geometry (Å, °)

*D*—H⋯*A*	*D*—H	H⋯*A*	*D*⋯*A*	*D*—H⋯*A*
O2—H2*A*⋯O4^i^	0.82	1.85	2.661 (2)	169
O3—H3*A*⋯O1^ii^	0.82	1.80	2.603 (2)	165
C15—H15⋯F1^iii^	0.93	2.48	3.382 (3)	163

Symmetry codes: (i) 
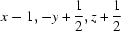
; (ii) 
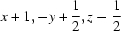
; (iii) 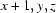.

**Fig. 1.**
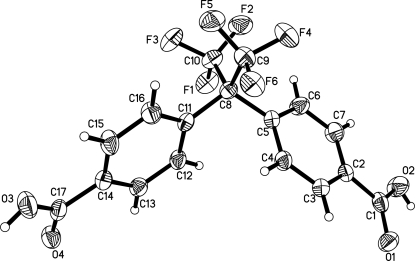
The molecular structure and labeling of (I). Displacement ellipsoids are drawn at the 30% probability level.

**Fig. 2.**
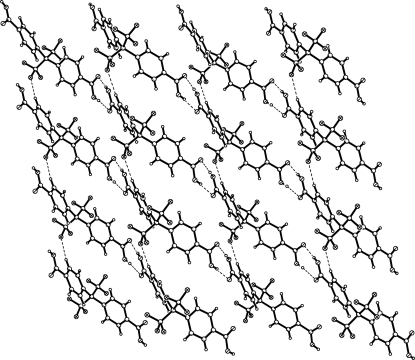
2D wave-like layer of (I). Dashed lines denote hydrogen bonds.

## supplementary crystallographic information

### Comment

The rational design and synthesis of novel solid-state architectures are of current interest in the field of supramolecular chemistry and crystal engineering, due to intriguing structural motifs that can be created by various intermolecular interactions. Supramolecular chemistry uses molecular recognition processes that rely heavily on the understanding of the recognition properties of the functional groups involved in these interactions (Zou *et al.*, 2007). Herein, we reported the organic crystal structure of C_17_H_10_F_6_O_4_, which is similar to that of the reported compounds (Potts *et al.* 2007; Lian *et al.*., 2007).

The molecular structure is shown in Fig. 1. The dihedral angle between the two benzene rings of the flexible H_2_hfipbb molecule is 67.43 (12)°. Strong intermolecular O—H···O and C—H···F hydrogen bonds (Table 1) link the molecules into the 2D wave-like layer structure (Fig. 2).

### Experimental

The title compound was prepared by hydrothermal method. A mixture of Zn(Ac)_2_.4H_2_O (0.20 mmol), 2,2'-bipyridine (bipy 0.20 mmol), 4,4'-(hexafluoroisopropylidene)bis(benzoic acid) (H_2_hfipbb 0.20 mmol) and water (10 ml) was stirred for 20 min. The mixture was then transferred to a 23 ml Teflon-lined autoclave and kept at 433 K for 72 h under autogenous pressure. Then the mixture was cooled to room temperature slowly, the targeted Zn complex was not obtained. Colorless single crystals of the title compound suitable for X-ray analysis were obtained from the reaction mixture.

### Refinement

H atoms were included in the riding approximation with C—H = 0.93 and O—H = 0.82 Å, U_iso_(H) = 1.2U_eq_(C,*O*).

### Crystal data

**Table:**

C_17_H_10_F_6_O_4_	*F*(000) = 792
*M**_r_* = 392.25	*D*_x_ = 1.593 Mg m^−^^3^
Monoclinic, *P*2_1_/*c*	Mo *K*α radiation, λ = 0.71073 Å
Hall symbol: -P 2ybc	Cell parameters from 775 reflections
*a* = 7.7523 (16) Å	θ = 2.7–18.7°
*b* = 13.381 (3) Å	µ = 0.16 mm^−^^1^
*c* = 16.134 (3) Å	*T* = 293 K
β = 102.294 (4)°	Prism, colorless
*V* = 1635.2 (6) Å^3^	0.35 × 0.20 × 0.18 mm
*Z* = 4	

### Data collection

**Table:**

Bruker SMART CCD diffractometer	2904 independent reflections
Radiation source: fine-focus sealed tube	1339 reflections with *I* > 2σ(*I*)
graphite	*R*_int_ = 0.053
φ and ω scans	θ_max_ = 25.1°, θ_min_ = 2.0°
Absorption correction: multi-scan (*SADABS*; Sheldrick, 1996)	*h* = −9→9
*T*_min_ = 0.963, *T*_max_ = 0.972	*k* = −15→15
8113 measured reflections	*l* = −13→19

### Refinement

**Table:**

Refinement on *F*^2^	Primary atom site location: structure-invariant direct methods
Least-squares matrix: full	Secondary atom site location: difference Fourier map
*R*[*F*^2^ > 2σ(*F*^2^)] = 0.040	Hydrogen site location: inferred from neighbouring sites
*wR*(*F*^2^) = 0.071	H-atom parameters constrained
*S* = 1.00	*w* = 1/[σ^2^(*F*_o_^2^) + (0.0135*P*)^2^ + 0.190*P*] where *P* = (*F*_o_^2^ + 2*F*_c_^2^)/3
2904 reflections	(Δ/σ)_max_ < 0.001
246 parameters	Δρ_max_ = 0.17 e Å^−^^3^
0 restraints	Δρ_min_ = −0.19 e Å^−^^3^

### Special details

**Table:**

Geometry. All esds (except the esd in the dihedral angle between two l.s. planes) are estimated using the full covariance matrix. The cell esds are taken into account individually in the estimation of esds in distances, angles and torsion angles; correlations between esds in cell parameters are only used when they are defined by crystal symmetry. An approximate (isotropic) treatment of cell esds is used for estimating esds involving l.s. planes.
Refinement. Refinement of *F*^2^ against ALL reflections. The weighted *R*-factor wR and goodness of fit *S* are based on *F*^2^, conventional *R*-factors *R* are based on *F*, with *F* set to zero for negative *F*^2^. The threshold expression of *F*^2^ > σ(*F*^2^) is used only for calculating *R*-factors(gt) etc. and is not relevant to the choice of reflections for refinement. *R*-factors based on *F*^2^ are statistically about twice as large as those based on *F*, and *R*- factors based on ALL data will be even larger.

### Fractional atomic coordinates and isotropic or equivalent isotropic displacement parameters (Å^2^)

**Table:**

	*x*	*y*	*z*	*U*_iso_*/*U*_eq_	
C1	−0.2346 (4)	0.0955 (2)	0.4514 (2)	0.0540 (8)	
C2	−0.1474 (3)	0.04538 (17)	0.38975 (18)	0.0467 (7)	
C3	0.0256 (3)	0.06770 (16)	0.38831 (17)	0.0482 (7)	
H3	0.0908	0.1094	0.4295	0.058*	
C4	0.1019 (3)	0.02807 (17)	0.32574 (17)	0.0491 (7)	
H4	0.2188	0.0435	0.3254	0.059*	
C5	0.0090 (3)	−0.03396 (17)	0.26363 (17)	0.0438 (7)	
C6	−0.1618 (3)	−0.05959 (18)	0.26780 (19)	0.0599 (8)	
H6	−0.2251	−0.1042	0.2286	0.072*	
C7	−0.2381 (3)	−0.01936 (19)	0.32963 (19)	0.0612 (8)	
H7	−0.3539	−0.0363	0.3309	0.073*	
C8	0.1010 (3)	−0.07485 (17)	0.19469 (18)	0.0430 (7)	
C9	0.2103 (4)	−0.1652 (2)	0.2347 (2)	0.0584 (8)	
C10	−0.0290 (4)	−0.1105 (2)	0.1152 (2)	0.0586 (8)	
C11	0.2132 (3)	0.00552 (17)	0.16376 (15)	0.0412 (7)	
C12	0.1452 (3)	0.10175 (17)	0.14883 (16)	0.0489 (7)	
H12	0.0372	0.1171	0.1621	0.059*	
C13	0.2346 (3)	0.17475 (18)	0.11477 (16)	0.0500 (7)	
H13	0.1856	0.2382	0.1045	0.060*	
C14	0.3958 (3)	0.15424 (18)	0.09593 (16)	0.0460 (7)	
C15	0.4658 (3)	0.05969 (19)	0.11157 (18)	0.0609 (8)	
H15	0.5758	0.0454	0.1002	0.073*	
C16	0.3744 (3)	−0.01372 (19)	0.14386 (17)	0.0575 (8)	
H16	0.4223	−0.0776	0.1524	0.069*	
C17	0.4938 (4)	0.2307 (2)	0.05836 (17)	0.0513 (8)	
O1	−0.1535 (2)	0.15916 (13)	0.50126 (12)	0.0654 (6)	
O2	−0.3969 (3)	0.07246 (13)	0.44691 (15)	0.0814 (7)	
H2A	−0.4415	0.1127	0.4745	0.098*	
O3	0.6508 (3)	0.20695 (12)	0.05373 (14)	0.0748 (6)	
H3A	0.7020	0.2563	0.0408	0.090*	
O4	0.4229 (2)	0.31255 (13)	0.03316 (12)	0.0628 (6)	
F1	−0.14945 (18)	−0.04108 (11)	0.08519 (10)	0.0701 (5)	
F2	−0.11835 (19)	−0.19326 (11)	0.12709 (10)	0.0760 (5)	
F3	0.05259 (19)	−0.13151 (11)	0.05246 (11)	0.0747 (5)	
F4	0.1158 (2)	−0.22673 (10)	0.27230 (11)	0.0775 (5)	
F5	0.2745 (2)	−0.22093 (10)	0.17918 (11)	0.0736 (5)	
F6	0.3482 (2)	−0.13628 (10)	0.29418 (11)	0.0664 (5)	

### Atomic displacement parameters (Å^2^)

**Table:**

	*U*^11^	*U*^22^	*U*^33^	*U*^12^	*U*^13^	*U*^23^
C1	0.054 (2)	0.0537 (18)	0.062 (3)	0.0027 (15)	0.0283 (19)	0.0126 (16)
C2	0.0535 (19)	0.0446 (15)	0.046 (2)	−0.0039 (14)	0.0196 (17)	0.0041 (14)
C3	0.0538 (18)	0.0478 (15)	0.044 (2)	−0.0051 (13)	0.0139 (17)	−0.0007 (14)
C4	0.0454 (17)	0.0559 (16)	0.049 (2)	−0.0061 (14)	0.0176 (17)	−0.0013 (15)
C5	0.0483 (18)	0.0442 (15)	0.041 (2)	−0.0032 (13)	0.0150 (16)	0.0021 (14)
C6	0.0547 (19)	0.0644 (17)	0.064 (3)	−0.0151 (14)	0.0211 (18)	−0.0206 (16)
C7	0.0477 (19)	0.0698 (19)	0.073 (3)	−0.0114 (15)	0.0275 (19)	−0.0068 (18)
C8	0.0423 (15)	0.0464 (15)	0.039 (2)	0.0000 (12)	0.0060 (15)	−0.0044 (14)
C9	0.055 (2)	0.0553 (19)	0.065 (3)	−0.0058 (16)	0.014 (2)	0.0039 (18)
C10	0.057 (2)	0.0604 (19)	0.059 (3)	0.0039 (16)	0.014 (2)	−0.0142 (18)
C11	0.0412 (16)	0.0499 (16)	0.0327 (19)	0.0042 (13)	0.0078 (14)	−0.0010 (13)
C12	0.0463 (17)	0.0543 (16)	0.051 (2)	0.0059 (13)	0.0216 (16)	0.0019 (15)
C13	0.0556 (18)	0.0479 (15)	0.049 (2)	0.0062 (14)	0.0162 (16)	0.0005 (14)
C14	0.0464 (17)	0.0536 (17)	0.039 (2)	0.0017 (14)	0.0107 (15)	0.0016 (14)
C15	0.0453 (17)	0.0694 (19)	0.076 (3)	0.0081 (15)	0.0307 (17)	0.0128 (17)
C16	0.0542 (19)	0.0532 (17)	0.070 (2)	0.0142 (14)	0.0240 (18)	0.0094 (16)
C17	0.0479 (19)	0.0664 (19)	0.044 (2)	−0.0031 (16)	0.0188 (17)	−0.0045 (16)
O1	0.0729 (14)	0.0669 (13)	0.0592 (16)	−0.0006 (10)	0.0205 (12)	−0.0136 (11)
O2	0.0789 (15)	0.0848 (15)	0.096 (2)	−0.0088 (12)	0.0523 (14)	−0.0270 (13)
O3	0.0644 (14)	0.0759 (14)	0.0945 (18)	−0.0037 (11)	0.0400 (13)	0.0129 (13)
O4	0.0660 (13)	0.0586 (11)	0.0685 (15)	0.0055 (10)	0.0252 (11)	0.0127 (11)
F1	0.0562 (10)	0.0868 (11)	0.0613 (13)	0.0170 (8)	−0.0013 (9)	−0.0071 (9)
F2	0.0732 (11)	0.0693 (10)	0.0835 (14)	−0.0196 (8)	0.0125 (10)	−0.0244 (10)
F3	0.0710 (11)	0.0992 (12)	0.0547 (12)	0.0075 (9)	0.0151 (10)	−0.0247 (9)
F4	0.0733 (11)	0.0613 (10)	0.1000 (16)	−0.0059 (8)	0.0231 (11)	0.0212 (9)
F5	0.0774 (12)	0.0578 (9)	0.0885 (15)	0.0155 (8)	0.0242 (11)	−0.0058 (9)
F6	0.0562 (10)	0.0689 (10)	0.0677 (13)	0.0017 (8)	−0.0013 (9)	0.0144 (9)

### Geometric parameters (Å, °)

**Table:**

C1—O1	1.246 (3)	C9—F5	1.340 (3)
C1—O2	1.283 (3)	C10—F1	1.333 (3)
C1—C2	1.477 (3)	C10—F3	1.332 (3)
C2—C7	1.377 (3)	C10—F2	1.341 (3)
C2—C3	1.379 (3)	C11—C16	1.379 (3)
C3—C4	1.380 (3)	C11—C12	1.393 (3)
C3—H3	0.9300	C12—C13	1.378 (3)
C4—C5	1.380 (3)	C12—H12	0.9300
C4—H4	0.9300	C13—C14	1.375 (3)
C5—C6	1.383 (3)	C13—H13	0.9300
C5—C8	1.544 (3)	C14—C15	1.379 (3)
C6—C7	1.374 (3)	C14—C17	1.479 (3)
C6—H6	0.9300	C15—C16	1.377 (3)
C7—H7	0.9300	C15—H15	0.9300
C8—C10	1.530 (3)	C16—H16	0.9300
C8—C11	1.532 (3)	C17—O4	1.254 (3)
C8—C9	1.538 (3)	C17—O3	1.276 (3)
C9—F4	1.331 (3)	O2—H2A	0.8200
C9—F6	1.333 (3)	O3—H3A	0.8200
			
O1—C1—O2	123.7 (3)	F6—C9—C8	111.0 (2)
O1—C1—C2	120.3 (3)	F5—C9—C8	114.0 (3)
O2—C1—C2	115.9 (3)	F1—C10—F3	106.3 (3)
C7—C2—C3	118.5 (3)	F1—C10—F2	106.5 (2)
C7—C2—C1	121.4 (3)	F3—C10—F2	106.1 (2)
C3—C2—C1	119.9 (3)	F1—C10—C8	111.9 (2)
C2—C3—C4	119.9 (2)	F3—C10—C8	111.6 (2)
C2—C3—H3	120.0	F2—C10—C8	113.9 (2)
C4—C3—H3	120.0	C16—C11—C12	117.4 (2)
C5—C4—C3	121.5 (2)	C16—C11—C8	123.4 (2)
C5—C4—H4	119.2	C12—C11—C8	119.0 (2)
C3—C4—H4	119.2	C13—C12—C11	121.4 (2)
C4—C5—C6	118.1 (2)	C13—C12—H12	119.3
C4—C5—C8	119.1 (2)	C11—C12—H12	119.3
C6—C5—C8	122.8 (2)	C14—C13—C12	120.3 (2)
C7—C6—C5	120.2 (3)	C14—C13—H13	119.9
C7—C6—H6	119.9	C12—C13—H13	119.9
C5—C6—H6	119.9	C13—C14—C15	118.9 (2)
C6—C7—C2	121.6 (2)	C13—C14—C17	121.5 (2)
C6—C7—H7	119.2	C15—C14—C17	119.5 (2)
C2—C7—H7	119.2	C14—C15—C16	120.6 (2)
C10—C8—C11	105.3 (2)	C14—C15—H15	119.7
C10—C8—C9	108.2 (2)	C16—C15—H15	119.7
C11—C8—C9	112.9 (2)	C11—C16—C15	121.3 (2)
C10—C8—C5	113.1 (2)	C11—C16—H16	119.3
C11—C8—C5	111.55 (19)	C15—C16—H16	119.3
C9—C8—C5	105.9 (2)	O4—C17—O3	123.8 (3)
F4—C9—F6	106.7 (3)	O4—C17—C14	120.6 (3)
F4—C9—F5	106.3 (2)	O3—C17—C14	115.6 (3)
F6—C9—F5	106.7 (2)	C1—O2—H2A	109.5
F4—C9—C8	111.7 (2)	C17—O3—H3A	109.5
			
O1—C1—C2—C7	−174.9 (2)	C11—C8—C10—F1	−70.1 (3)
O2—C1—C2—C7	2.1 (4)	C9—C8—C10—F1	168.9 (2)
O1—C1—C2—C3	1.2 (4)	C5—C8—C10—F1	51.9 (3)
O2—C1—C2—C3	178.2 (2)	C11—C8—C10—F3	48.8 (3)
C7—C2—C3—C4	2.2 (4)	C9—C8—C10—F3	−72.2 (3)
C1—C2—C3—C4	−173.9 (2)	C5—C8—C10—F3	170.8 (2)
C2—C3—C4—C5	0.1 (4)	C11—C8—C10—F2	169.0 (2)
C3—C4—C5—C6	−3.0 (4)	C9—C8—C10—F2	48.0 (3)
C3—C4—C5—C8	179.2 (2)	C5—C8—C10—F2	−69.0 (3)
C4—C5—C6—C7	3.6 (4)	C10—C8—C11—C16	−94.9 (3)
C8—C5—C6—C7	−178.7 (2)	C9—C8—C11—C16	22.9 (4)
C5—C6—C7—C2	−1.3 (4)	C5—C8—C11—C16	142.0 (2)
C3—C2—C7—C6	−1.6 (4)	C10—C8—C11—C12	80.3 (3)
C1—C2—C7—C6	174.5 (3)	C9—C8—C11—C12	−161.9 (2)
C4—C5—C8—C10	−159.9 (2)	C5—C8—C11—C12	−42.7 (3)
C6—C5—C8—C10	22.4 (3)	C16—C11—C12—C13	0.6 (4)
C4—C5—C8—C11	−41.5 (3)	C8—C11—C12—C13	−174.9 (2)
C6—C5—C8—C11	140.9 (2)	C11—C12—C13—C14	−1.1 (4)
C4—C5—C8—C9	81.7 (3)	C12—C13—C14—C15	0.1 (4)
C6—C5—C8—C9	−95.9 (3)	C12—C13—C14—C17	179.3 (2)
C10—C8—C9—F4	−73.2 (3)	C13—C14—C15—C16	1.3 (4)
C11—C8—C9—F4	170.6 (2)	C17—C14—C15—C16	−177.8 (3)
C5—C8—C9—F4	48.3 (3)	C12—C11—C16—C15	0.9 (4)
C10—C8—C9—F6	167.9 (2)	C8—C11—C16—C15	176.1 (3)
C11—C8—C9—F6	51.7 (3)	C14—C15—C16—C11	−1.9 (4)
C5—C8—C9—F6	−70.7 (3)	C13—C14—C17—O4	−8.7 (4)
C10—C8—C9—F5	47.3 (3)	C15—C14—C17—O4	170.4 (3)
C11—C8—C9—F5	−68.9 (3)	C13—C14—C17—O3	171.5 (3)
C5—C8—C9—F5	168.8 (2)	C15—C14—C17—O3	−9.4 (4)

### Hydrogen-bond geometry (Å, °)

**Table:**

*D*—H···*A*	*D*—H	H···*A*	*D*···*A*	*D*—H···*A*
O2—H2A···O4^i^	0.82	1.85	2.661 (2)	169
O3—H3A···O1^ii^	0.82	1.80	2.603 (2)	165
C15—H15···F1^iii^	0.93	2.48	3.382 (3)	163

Symmetry codes: (i) *x*−1, −*y*+1/2, *z*+1/2; (ii) *x*+1, −*y*+1/2, *z*−1/2; (iii) *x*+1, *y*, *z*.
